# The Streptomyces filipinensis Gamma-Butyrolactone System Reveals Novel Clues for Understanding the Control of Secondary Metabolism

**DOI:** 10.1128/AEM.00443-20

**Published:** 2020-09-01

**Authors:** Eva G. Barreales, Tamara D. Payero, Ester Jambrina, Jesús F. Aparicio

**Affiliations:** a Area of Microbiology, Faculty of Biology, University of León, León, Spain; University of California, Davis

**Keywords:** ARE sequence, filipin, GBL, GBL pseudo-receptor, GBL receptor, polyene macrolide, *Streptomyces*

## Abstract

*Streptomyces* γ-butyrolactones (GBLs) are quorum sensing communication signals triggering antibiotic production. The GBL system of Streptomyces filipinensis, the producer of the antifungal agent filipin, has been investigated. Inactivation of *sfbR* (for S. filipinensis γ-butyrolactone receptor), a GBL receptor, resulted in a strong decrease in production of filipin, and deletion of *sfbR2*, a pseudo-receptor, boosted it, in agreement with lower and higher levels of transcription of filipin biosynthetic genes, respectively. It is noteworthy that none of the mutations affected growth or morphological development. While no ARE (autoregulatory element)-like sequences were found in the promoters of filipin genes, suggesting indirect control of production, five ARE sequences were found in five genes of the GBL cluster, whose transcription has been shown to be controlled by both S. filipinensis SfbR and SfbR2. *In vitro* binding of recombinant SfbR and SfbR2 to such sequences indicated that such control is direct. Transcription start points were identified by 5′ rapid amplification of cDNA ends, and precise binding regions were investigated by the use of DNase I protection studies. Binding of both regulators took place in the promoter of target genes and at the same sites. Information content analysis of protected sequences in target promoters yielded an 18-nucleotide consensus ARE sequence. Quantitative transcriptional analyses revealed that both regulators are self-regulated and that each represses the transcription of the other as well as that of the remaining target genes. Unlike other GBL receptor homologues, SfbR activates its own transcription whereas SfbR2 has a canonical autorepression profile. Additionally, SfbR2 was found here to bind the antifungal antimycin A as a way to modulate its DNA-binding activity.

**IMPORTANCE** *Streptomyces* GBLs are important signaling molecules that trigger antibiotic production in a quorum sensing-dependent manner. We have characterized the GBL system from S. filipinensis, finding that two key players of this system, the GBL receptor and the pseudo-receptor, each counteracts the transcription of the other for the modulation of filipin production and that such control over antifungal production involves an indirect effect on the transcription of filipin biosynthetic genes. Additionally, the two regulators bind the same sites, are self-regulated, and repress the transcription of three other genes of the GBL cluster, including that encoding the GBL synthase. In contrast to all the GBL receptors known, SfbR activates its own synthesis. Moreover, the pseudo-receptor was identified as the receptor of antimycin A, thus extending the range of examples supporting the idea of signaling effects of antibiotics in *Streptomyces*. The intricate regulatory network depicted here should provide important clues for understanding the regulatory mechanism governing secondary metabolism.

## Abstract (precis)

*Streptomyces* GBLs are important signaling molecules that trigger antibiotic production in a quorum sensing-dependent manner. We have characterized the GBL system from S. filipinensis, finding that two key players of this system, the GBL receptor and the pseudo-receptor, each counteracts the transcription of the other for the modulation of filipin production and that such control over antifungal production involves an indirect effect on the transcription of filipin biosynthetic genes. Additionally, the two regulators bind the same sites, are self-regulated, and repress the transcription of three other genes of the GBL cluster, including that encoding the GBL synthase. In contrast to all the GBL receptors known, SfbR activates its own synthesis. Moreover, the pseudo-receptor was identified as the receptor of antimycin A, thus extending the range of examples supporting the idea of signaling effects of antibiotics in *Streptomyces*. The intricate regulatory network depicted here should provide important clues for understanding the regulatory mechanism governing secondary metabolism.

## INTRODUCTION

*Streptomyces* are soil-dwelling bacteria that undergo a rather complex differentiation process which is usually accompanied by production of antibiotic and other secondary metabolite-bioactive molecules such as anticancer agents, immunosuppressants, and antihelminthic agents, among others (1). The onset of this process is often controlled by small extracellular signaling molecules (autoregulators) that coordinate the population behavior at nanomolar concentrations; hence, they have sometimes been regarded as bacterial hormones. Several classes of autoregulators have been identified in *Streptomyces*, including furanes (2), butenolides (3), butanediols (4), and diketopiperazines (5), but the most thoroughly studied group is that of the γ-butyrolactones (GBLs). These share a 2,3-disubstituted GBL scaffold with a variable C-2 side chain which is species specific (6) except for one example, SVB1 from S. venezuelae, which is identical to SCB3 from S. coelicolor (7).

GBLs elicit secondary metabolite biosynthesis by regulating the DNA-binding activity of cognate receptor proteins. In a normal scenario, the GBL interacts with its specific receptor protein and releases its repression of target genes, thus activating gene expression (6). Target genes are generally involved in secondary metabolism but occasionally also in morphological differentiation (8). Most GBL receptors target cluster-situated regulatory genes linked to secondary metabolite gene clusters or the global regulatory gene *adpA* (9); hence, given the wide influence of such regulators (10, 11), effects on gene expression in most cases represent results of activation of regulatory cascade mechanisms (12). Although most GBL receptors act as transcriptional repressors (13), some, such as SpbR from S. pristinaespiralis (14) and SprA from S. chattanoogensis (15), have been described to act as positive regulators, whereas others, such as JadR3 in S. venezuelae (7), have both repressor and activator activities depending on the availability of GBL.

Our knowledge concerning GBL biosynthesis is limited, but it seems clear that a protein homologous to AfsA, the key enzyme in A-factor biosynthesis in S. griseus, is required for the formation of GBLs (16, 17). AfsA catalyzes the first step of the biosynthesis, the condensation of dihydroxyacetone phosphate (a glycerol derivative) and a β-ketoacid to form a fatty acid ester, which is converted into A-factor by the three steps of dephosphorylation, aldol condensation, and reduction (18, 19).

In *Streptomyces* genomes, *afsA*-like genes are commonly found next to or near GBL receptor-encoding genes and located in the vicinity of or within antibiotic biosynthetic gene clusters. In general, the receptor acts as a repressor of the biosynthesis of its specific GBL synthase and regulates its own synthesis, forming a negative-feedback loop, in addition to modulating the secondary metabolism (9). Many *Streptomyces* spp. additionally possess a range of auxiliary regulators, harbored within the same GBL gene cluster, that modulate the activity of this central circuit (20). Among such auxiliary regulators are pseudo-receptors, homologues of GBL receptor proteins without the ability to bind the GBL ligand but with the capacity to bind other ligands, such as antibiotics (21–23), and with a role in the regulation of GBL production (23–25).

S. filipinensis produces a family of polyene polyketide macrolides known as filipins, which have broad-spectrum antifungal activity, with filipin III being the major component. Although filipin III has been reported to be produced by other strains, S. filipinensis is the species used for industrial production of the antifungal, with its production being substantially higher than in other strains (26). Despite being a polyene and having potent antifungal activity derived from its interaction with the ergosterol of fungal membranes, this pentaene shows a rather high affinity for cholesterol, which makes it useless in human therapy. Nonetheless, it is widely used for detection and the quantitation of cholesterol in biological membranes (27) and as a tool for diagnosis of Niemann-Pick type C disease (28). Its biosynthetic pathway in S. filipinensis has recently been discovered (26), and, other than a recent study on the mechanism of phosphate control of filipin biosynthesis (29), there is an absolute lack of knowledge of the regulatory mechanisms of antibiotic production in this bacterium. It was therefore of great interest to study the GBL system of S. filipinensis and its role on filipin production.

## RESULTS AND DISCUSSION

### Cloning of a γ-butyrolactone gene cluster in S. filipinensis.

**FIG 1:**
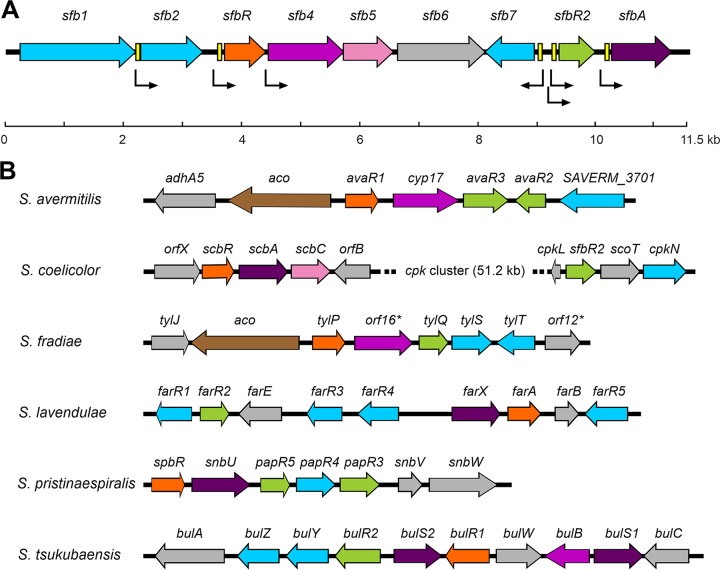
GBL gene cluster from S. filipinensis and other *Streptomyces* species. (A) The pointed boxes indicate the direction of transcription. The locations of identified ARE sequences are indicated by yellow boxes. (B) Genetic organization of GBL clusters in several *Streptomyces* species. The GBL receptor gene is marked in orange and the pseudo-receptor in green. Genes coding for other regulators are shaded in blue, while those genes that might be involved in the GBL biosynthesis are in purple (AfsA-like protein), violet (P450 monooxygenase), pink (dehydrogenase), or brown (Acyl-CoA oxidase). Deduced transcriptional units are indicated by arrows.

The GBL gene cluster was identified by hybridization using a cosmid library (26) and a 149-bp probe obtained by PCR amplification of S. filipinensis chromosomal DNA with degenerate oligonucleotides derived from conserved stretches of the N-terminal region of several GBL receptors (see Materials and Methods). Once a gene homologous to those encoding GBL receptors was identified (*sfbR* [for S. filipinensis γ-butyrolactone receptor]), the remaining genes of the cluster were identified by chromosome walking. The deduced gene organization within this region is shown in Fig. 1A.

### *In silico* analysis and arrangement of genes.

Computer-assisted analysis of the 11,542-bp sequenced region revealed nine complete open reading frames (ORFs). Among these genes, two GBL receptor-like-encoding genes were identified and named *sfbR* and *sfbR2* and a gene homologous to GBL synthase-encoding genes was identified and named *sfbA*. Table S1 in the supplemental material shows the deduced functions of all these genes.

At the left end of the cluster, *sfb1* encodes a large-sized SARP (*Streptomyces* antibiotic regulatory protein)-like regulator showing the same domain architecture as AfsR from S. coelicolor (30) and 39% identity along its full length. *sfb2*, which encodes a putative regulator of the StrR family showing 49% identity with KasT, a regulator encoded by the kasugamycin biosynthetic cluster of *S. kasugaensis* (31), lies downstream and in the same orientation. Both *sfb2* and *kasT* contain the rare leucine TTA codon, which has been correlated with regulatory genes involved in antibiotic production (32). *sfbR* is situated downstream and encodes a protein with convincing similarity to GBL receptors from other *Streptomyces* spp. The highest scores were seen with SpbR from S. pristinaespiralis (14) (57% identity) and AvaR1 from S. avermitilis (3) (56% identity). An alignment with other GBL receptors is shown in Fig. S1 in the supplemental material.

Downstream from *sfbR* is *sfb4*, a cytochrome P450 monooxygenase-encoding gene. It shows highest similarity to SnbU from S. pristinaespiralis (33) (58% identity), Orf16* from S. fradiae (34) (57% identity), and Cyp17 from S. avermitilis (35) (56% identity). Interestingly, in all these four cases the genes for cytochrome monooxygenases are adjacent to genes coding for GBL receptors, suggesting that these enzymes may be involved in the biosynthesis of butyrolactone, although their role has been demonstrated only in Cyp17, which is implicated in the biosynthesis of avenolide, a signaling molecule of the butenolide type (3). *sfb4* also contains the rare leucine TTA codon, a feature that it shares with other cytochrome P450 monooxygenase-encoding genes from GBL gene clusters, such as orf16* from S. fradiae (34) and *tsuB* from S. tsukubaensis (36). *sfb5* is located downstream; it encodes a putative short-chain dehydrogenase/reductase with similarity to the Orf4 protein from *Streptomyces* sp. strain SBI034 (37) (51% identity) and ScbC from S. coelicolor (38) (50% identity). These enzymes have been proposed to be nucleoside diphosphate-sugar epimerases involved in GBL biosynthesis; thus, it is conceivable that S. filipinensis Sfb5 could have a role in the biosynthesis of S. filipinensis GBL. The two genes that follow are oriented convergently (Fig. 1A): *sfb6* encodes a hypothetical protein of unknown function, and *sfb7*, whose coding strand is opposite all the remaining identified genes, encodes a small-sized SARP-like regulator. This protein is highly similar to other SARPs belonging to GBL clusters, such as BulY from S. tsukubaensis (36), FarR4 from S. lavendulae (39), and SgvR2 from S. griseoviridis (40) (66%, 65%, and 65% identity, respectively).

Downstream from *sfb7* is *sfbR2* (Fig. 1A), which encodes a putative GBL pseudo-receptor (see below). It showed the highest similarity scores to PapR5 from S. pristinaespiralis (33) (51% identity) and TylQ from S. fradiae (34) (47% identity). An alignment of SfbR2 with other GBL pseudoreceptors is shown in Fig. S2. Downstream and in the same orientation lies *sfbA*, whose product shows high similarity to GBL synthases such as SrrX from S. rochei (41) (57% identity) and Lct9 from S. rishiriensis (42) (55% identity). An alignment of SfbA with other GBL synthases is shown in Fig. S3.

No obvious synteny with other GBL gene clusters is observed. Although the genes encoding the GBL receptor and pseudo-receptor tend to be clustered with the GBL biosynthesis gene(s) (6) (Fig. 1B), many exceptions have been described. Thus, S. avermitilis or S. fradiae GBL gene clusters lack GBL synthase-encoding genes and harbor an acyl coenzyme A (acyl-CoA) oxidase-encoding gene (*aco*) instead that possibly is capable of replacing the GBL synthase's role (3, 34). Another exception is the S. coelicolor GBL gene cluster, where the *scbR2* pseudo-receptor gene is separated from the GBL synthase and the receptor genes by the coelimycin *cpk* cluster (Fig. 1B) (43). Hence, it seems that there are no unifying principles among GBL gene clusters.

### SfbR is a putative GBL receptor whereas SfbR2 is a pseudo-receptor.

Both the GBL receptors and pseudo-receptors belong to the TetR family of regulators. These comprise a conserved helix-turn-helix DNA-binding motif at the N-terminal region and a variable C-terminal domain involved in ligand binding (44, 45). The latter contains a highly conserved tryptophan residue which is involved in ligand binding (46). Interestingly, SfbR and SfbR2 share 34% identity and both contain such conserved tryptophan residues (W123 in SfbR and W128 in SfbR2 [Fig. S1 and S2]).

**FIG 2:**
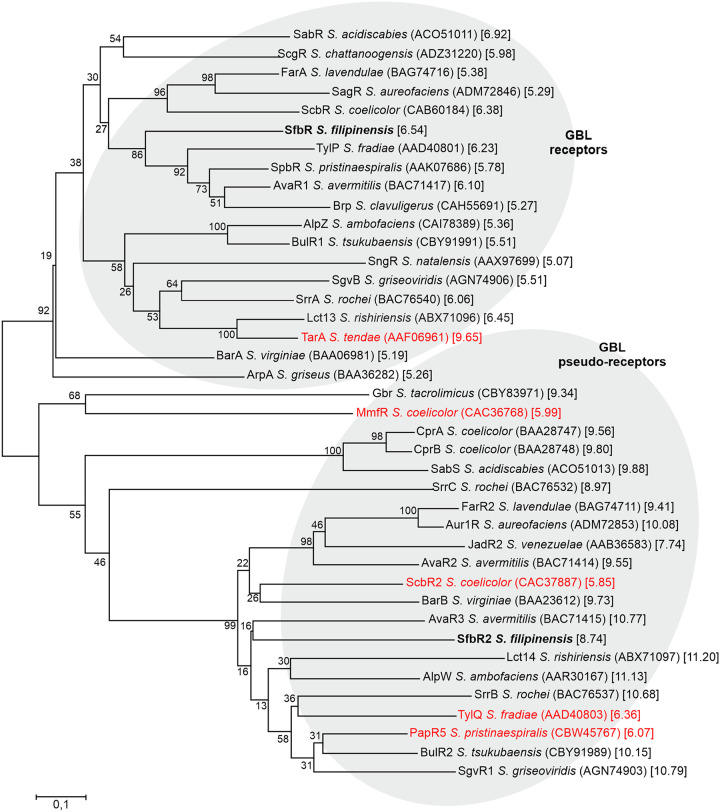
SfbR and SfbR2 phylogenetic tree. Homologues used for phylogenetic analyses were chosen randomly among those that were well characterized and belonged to representative *Streptomyces* species. The tree was constructed using the neighbor-joining method. NCBI accession numbers are indicated between brackets, and calculated pI values are shown between square brackets. Proteins that showed atypical pI values are in red. The reliability of each node was analyzed by the bootstrap test with 1,000 replicates, and the percentage obtained is indicated. The bar indicates 0.1 substitution per amino acid position.

Despite their structural similarity, compared with counterparts in the databases, SfbR clusters with genuine GBL receptors whereas SfbR2 does so with pseudo-receptors. Results of a phylogenetic analysis performed with sequences of whole amino acids are shown in Fig. 2. In addition, SfbR has a calculated pI of 6.54, a slightly acidic-neutral value that is characteristic of genuine receptors, whereas that of SfbR2 is 8.74, a basic value common for pseudo-receptors (39). Most of the proteins analyzed follow such a paradigm, but there are a few exceptions (indicated in red in Fig. 2). It is noteworthy that MmfR, an authentic methylenomycin furan receptor from S. coelicolor (47), has a pI value of 5.99, as expected, but clusters with pseudo-receptors and that ScbR2, an S. coelicolor GBL pseudo-receptor (21), clusters with pseudo-receptors, as expected, but has a slightly acidic pI value of 5.85.

### Inactivation of *sfbR* reduces filipin production whereas *sfbR2* deletion increases it.

To assess the functions of *sfbR* and *sfbR2*, we deleted them by using the REDIRECT gene replacement technology as indicated in Materials and Methods. Double-crossover mutants that had lost *sfbR* were screened by apramycin resistance analysis, whereas those lacking *sfbR2* were selected by their resistance to spectinomycin. All mutants were further verified by PCR analysis (Fig. S4).

**FIG 3:**
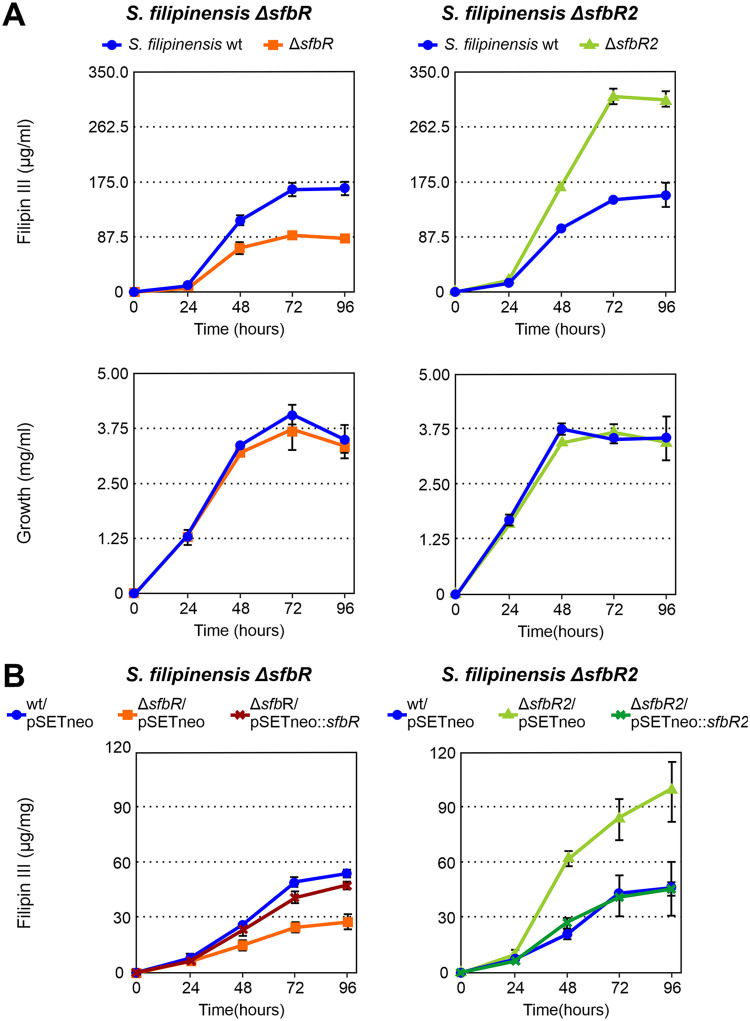
SfbR inactivation decreases filipin production, and SfbR2 deletion increases it. (A) Time course quantification of filipin III production and growth curves in the wild-type and mutant strains. Fermentations were carried out at 30°C in YEME medium. (B) Effects of gene complementation in YEME medium. Growth curves were identical in all cases. Data represent averages of results from three duplicate flasks. Vertical bars indicate standard deviations of the mean values.

In order to study the effect that the inactivation of the *sfbR* and *sfbR2* genes had on the production of filipin, fermentation broths produced by the new mutant strains, grown in yeast extract-malt extract (YEME) medium, were extracted with methanol and analyzed for the presence of filipin III (the major component of the filipin complex). Results indicated that S. filipinensis Δ*sfbR* was impaired in filipin production, reaching ca. 50% of the production observed in the parental strain (Fig. 3A), thus suggesting that SfbR is an activator of filipin biosynthesis. In contrast, S. filipinensis Δ*sfbR2* behaved as a filipin overproducer, producing filipin at about 2-fold the level produced by the wild-type strain at 72 h of growth (Fig. 3A), which suggested that SfbR2 is a negative regulator of antifungal biosynthesis. Interestingly, neither of the mutations affected growth or morphological development on solid medium (not shown), and the growth curves of the mutants closely resembled the growth of their parental strain (Fig. 3A). This indicates that neither SfbR nor SfbR2 significantly affects primary metabolism under the conditions used.

### Gene complementation restores filipin biosynthesis in the mutants.

To confirm that the gene deletions were directly responsible for the observed effects on filipin III production, we complemented both mutants with the corresponding genes. For that purpose, we introduced one copy of the gene, including its promoter region, into the genome of the Δ*sfbR* and Δ*sfbR2* mutants using integrative plasmids pSETneo::sfbR and pSETneo::sfbR2, respectively (see Materials and Methods). pSETneo (48) was also introduced into the parental strain as a control. No differences between the complemented strains and the control were observed with respect to growth.

Introduction of a copy of *sfbR* into S. filipinensis Δ*sfbR* boosted its ability to produce filipin III, almost restoring it to the levels seen with the parental strain, whereas introduction of *sfbR2* into S. filipinensis Δ*sfbR2* reduced its ability to produce the antifungal at the same levels as were seen with the wild-type strain (Fig. 3B). These results indicate that the two regulators control filipin biosynthesis in opposite ways.

Counteraction between GBL receptor and pseudo-receptor is not uncommon in *Streptomyces* spp. In S. pristinaespiralis, SpbR activates pristinamycin production whereas both the PapR3 and PapR5 pseudo-receptors repress its production (14, 49). Similarly, in S. aureofaciens, GBL receptor SagR and pseudo-receptor Aur1R behave as an auricin biosynthesis activator and repressor, respectively (25, 50). The same type of competition has also been described previously in S. venezuelae (7, 21).

### SfbR and SfbR2 control expression of *fil* genes indirectly.

In order to study whether the effect on filipin production in the mutants was a direct consequence of a higher or lower level of transcription of filipin biosynthetic genes compared to the parental strain, we performed gene expression studies by reverse transcription-quantitative PCR (RT-qPCR). Total RNA was prepared from cultures after growth for 48 h in YEME medium without sucrose and used for analysis. The transcriptional levels of selected genes corresponding to different operons governing filipin biosynthesis in the mutant strains were compared with those measured in the wild-type strain, to which a relative expression value of 1 was assigned. The genes selected included polyketide synthase genes *filA1* and *filA2*, the thioesterase-encoding *filH* gene, and two cluster-situated regulator genes (*filR* and *filF*) (26).

**FIG 4:**
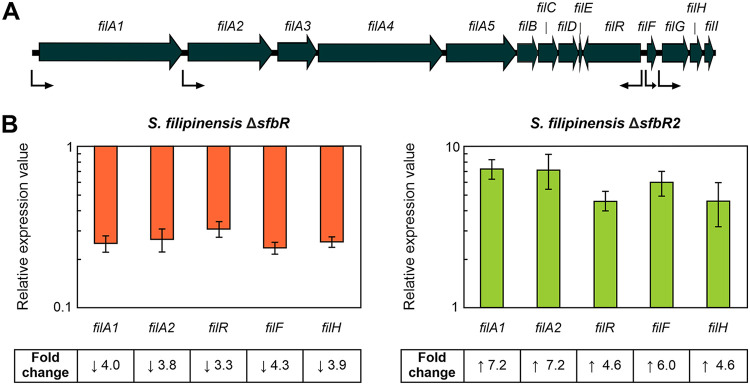
Gene expression analysis of filipin biosynthetic genes in the mutant strains. (A) Filipin biosynthetic gene cluster. Transcriptional units (26) are indicated by bent arrows. (B) Transcription was assessed by RT-qPCR. Total RNA was prepared after growth for 48 h in YEME medium without sucrose. Fold change values are relative to the parental strain's gene expression level, which was set to 1. The expression of *rrnA1* (encoding 16S rRNA) was used as a control. Error bars were calculated by measuring the standard deviations of the ratio values among three biological and three technical replicates of each sample. Fold change values are indicated below the panels. Primers are listed in Table 1.

In agreement with the overproduction of filipin in the Δ*sfbR2* mutant and the decreased production of filipin in the Δ*sfbR* mutant, all the selected genes showed the same pattern of expression, i.e., overexpression in the Δ*sfbR2* mutant and repression in the Δ*sfbR* mutant (Fig. 4), which indicates that SfbR2 is a repressor and SfbR an activator of filipin production.

In the absence of their ligands, GBL receptors recognize and bind to palindromic sequences rich in adenine and thymine, called AREs (autoregulatory elements), present in the promoter regions of target genes (in many cases, representing their own encoding genes), repressing them (14, 20, 51, 52). Recent studies have shown that pseudo-receptors are also capable of recognizing and binding to the same operator sequences (24, 25, 36, 53). Bioinformatic analysis of the sequence of intergenic regions within the *fil* cluster revealed no ARE sequences, thus suggesting that neither SfbR nor SfbR2 can bind to these regions and that the control exerted by either regulator must be indirect and must operate via a second transcriptional regulator(s).

In contrast to what we have observed in S. filipinensis, in most of the cases reported, the control of secondary metabolite biosynthesis takes place directly by binding of the receptor and/or pseudo-receptor to ARE sequences at the promoters of key genes of secondary metabolite gene clusters. Such control has been described for ScbR and ScbR2, which control coelimycin biosynthesis in S. coelicolor (21, 54); AvaR1, AvaR2, and AvaR3, which regulate avermectin production in S. avermitilis (23, 55, 56); SpbR, PapR3, and PapR5, controlling pristinamycin biosynthesis in S. pristinaespiralis (14, 49); and JadR2 and JadR3, which control jadomycin production in S. venezuelae (7, 21), among others. However, a case similar to that of the S. filipinensis paradigm can be found in S. chattanoogensis, where SprA, a GBL receptor, stimulates transcription of several pimaricin biosynthetic genes and antifungal production in an indirect manner (15).

### Organization of transcriptional units within the GBL cluster.

To obtain an overall picture of the transcriptional arrangement of the *sfb* genes in S. filipinensis, it was necessary to determine the operons governing their transcription. Because of their divergent locations, the *sfb7* and *sfbR2* genes must have their own promoters. As for the rest of the genes of the group, to analyze the possible coupled transcription of neighboring genes, we performed RT-PCR using RNA from 48-h mycelia. These analyses detected transcripts containing the intergenic regions between *sfb4* and *sfb5* and between *sfb5* and *sfb6* (Fig. S5), thus suggesting that these genes could constitute an operon. Similarly, we detected a transcript containing the intergenic region between *sfbR2* and *sfbA*, whereas no amplification was observed between *sfb1* and *sfb2*, between *sfb2* and *sfbR*, or between *sfbR* and *sfb4* (Fig. S5). These results indicate that *sfbA* can be transcribed as part of a bicistronic transcript from the *sfbR2* promoter whereas *sfb2*, *sfbR* and *sfb4* must have their own promoters (see below) (Fig. 1A).

### Characterization of promoters of the GBL gene cluster containing ARE sequences.

**FIG 5:**
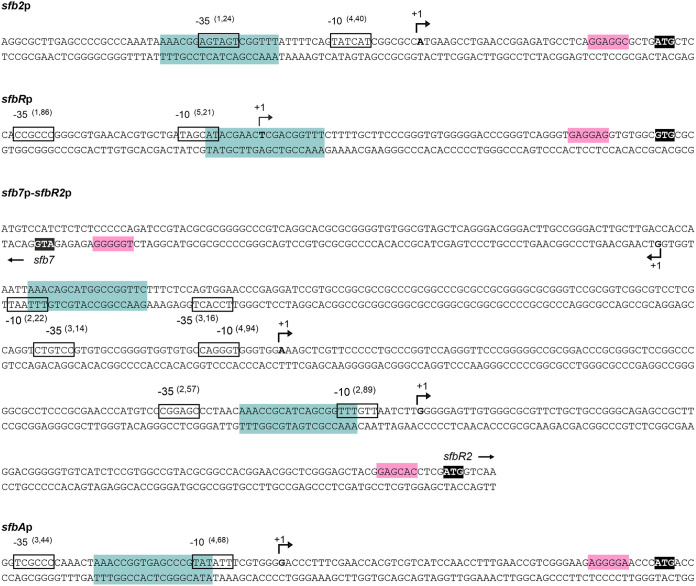
The promoters of some genes of the GBL cluster contain ARE sequences. The position of the transcriptional start point was determined by 5′ RACE. The putative −10 and −35 hexanucleotides are boxed. Scores resulting from the comparison to the matrices performed as reported previously by Bourn and Babb (57) for *Streptomyces* are indicated between brackets. The TSP is indicated by a bent arrow and bold letters. Nucleotides showing homology with the 16S RNA and which could form a ribosome-binding site are shaded in pink. Putative ARE sequences are shaded in blue. Start codons are highlighted in black.

Analysis of the GBL gene cluster revealed five possible ARE sequences, located in the upstream regions of genes *sfb2*, *sfbR*, *sfb7*, *sfbR2*, and *sfbA* (Fig. 1A and 5). To assess whether those regions constituted real promoters, we determined the transcriptional start points (TSPs) of those genes by 5′ rapid amplification of cDNA ends (5′ RACE). The corresponding −10 and −35 boxes of each promoter were established by comparison with the matrices reported previously by Bourn and Babb (57) for *Streptomyces* that take into account the nucleotides occurring in 13-nucleotide stretches, including the −10 or −35 consensus hexamers (see Materials and Methods). Results are summarized in Fig. 5.

The *sfb2* TSP is located at an adenine 35 nucleotides upstream of the ATG start codon. Analysis of the upstream sequence revealed TATCAT and AGTAGT to be the −10 and −35 boxes. The two boxes are separated by 14 nucleotides, with the −10 hexamer centered at 10 nucleotides from the TSP (Fig. 5). The TSP of *sfbR* is located at a thymine 59 nucleotides upstream from the GTG start codon. The sequence TAGCAT, centered at position −9, constitutes the −10 consensus, and a −35 box CCGCCC was identified at a distance of 19 nucleotides. In the case of *sfb7*, the TSP was identified at a cytosine 84 nucleotides upstream from the ATG. The −10 and −35 boxes (TTTAAT and TCCACT, respectively) were centered at positions −9 and −37 nucleotides from the TSP and were separated by 22 nucleotides (Fig. 5). For its part, *sfbR2* presented two TSPs, one at an adenine and the second one at a guanine located 234 and 108 nucleotides, respectively, upstream from the ATG start codon. The one at position −234 corresponds to a promoter with −10 (CAGGGT) and −35 (CTGTCC) boxes separated by 19 nucleotides, while the one at position −108 is controlled by a promoter with −10 and −35 boxes (TTTGTT and CGGAGC, respectively) separated by 21 nucleotides (Fig. 5). Finally, the TSP of *sfbA* was identified at a guanine 56 nucleotides upstream from the ATG codon. The analysis of the upstream sequence revealed a clear promoter, with the −10 box TATATT located 10 nucleotides upstream from the observed TSP and the −35 box TCGCCC separated by 21 nucleotides (Fig. 5). This finding, together with previous results, indicates that *sfbA* can be transcribed as a monocistronic transcript from its own promoter and as a bicistronic transcript from the *sfbR2* promoter.

Interestingly, the putative ARE sequence of *sfb2* promoter overlapped the −35 box whereas the remaining ARE sequences overlapped −10 boxes of the promoters studied (Fig. 5). The significance of this finding is unclear.

Taken together, these results suggest that S. filipinensis may exhibit a rather more complex form of control of GBL genes than other *Streptomyces* spp. On one side, while two promoters could direct the transcription of the bicistronic *sfbR2-sfbA* mRNA, only one of them (the one that is closer to the translation start) contains an ARE box. This suggests that these genes could partially avoid self-regulation while being transcribed from the distal promoter. On the other side, *sfbA* also has its own dedicated promoter which contains an ARE box. This feature suggests that *sfbA* transcription could have various points of control by SfbR or SfbR2 or both.

### Transcription of the γ-butyrolactone gene cluster is controlled by both SfbR and SfbR2.

In order to examine the roles of SfbR and SfbR2 regulators in the transcription of the genes whose promoters contained ARE sequences, we measured gene expression in the mutants by RT-qPCR. Total RNAs obtained from 24-, 48-, and 72-h cultures were used as templates, and the transcriptional levels of each gene in the different strains were compared with those of the parental strain, which was assigned a relative expression value of 1.

**FIG 6:**
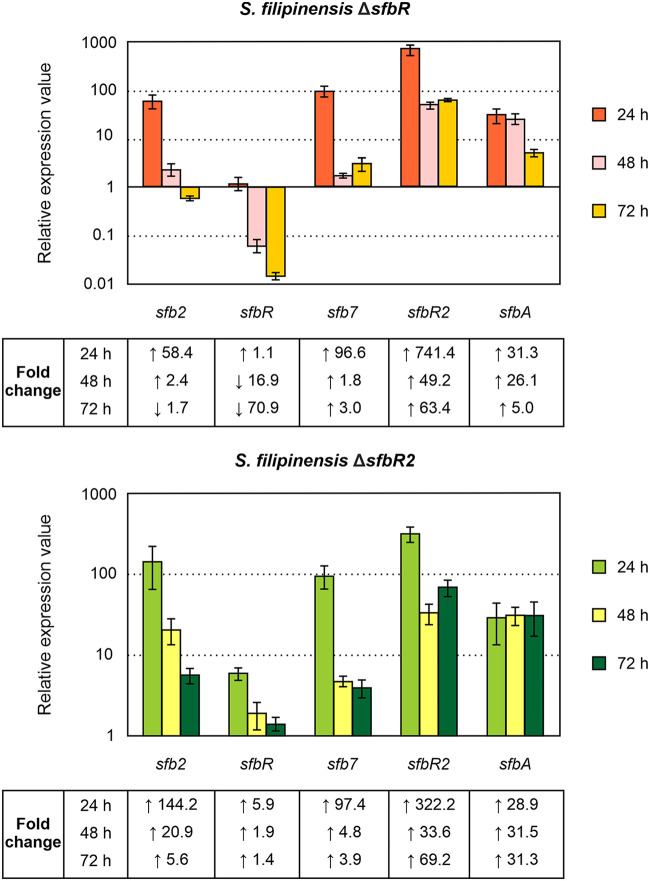
Analysis of expression of *sfb* genes in the mutants. Transcription was assessed by RT-qPCR. Fold change values are relative to the parental strain's gene expression level, which was set to 1. The level of expression of *rrnA1* (encoding 16S rRNA) was used as a control. Error bars were calculated by determining the standard deviations of the ratios of values among three biological and three technical replicates of each sample. The RNA templates were from 24-h, 48-h, and 72-h cultures grown in YEME medium without sucrose. Fold change values are indicated below the panels. Primers are listed in Table 1.

In order to assess the transcription of the deleted genes, primers were designed to generate PCR products near the 5′ end of the mRNA. Interestingly, transcription of the GBL genes was controlled by both SfbR and SfbR2. The absence of SfbR2 caused an increase in the transcription of all the genes studied, including that of its own gene, which indicates that it behaved as a repressor of all these genes, in particular at 24 h. In contrast, the absence of SfbR caused an increase in the levels of transcription of all the genes except for that of its own gene, which was reduced (Fig. 6). This result indicates that SfbR is an activator of its own synthesis and a repressor of the remaining genes studied. The self-activation of *sfbR* transcription was completely unexpected since GBL receptors normally act as repressors of their own synthesis (3, 23, 36, 39).

In GBL regulatory systems, it is common for receptors and pseudo-receptors to repress synthase expression (7, 15, 24, 53, 58) such that the GBL accumulates very slowly until it reaches a critical concentration, at which time it binds the receptor and releases it from target promoters. According to our results, S. filipinensis follows such a general model in which the GBL receptors and pseudo-receptors act as repressors of the synthase gene (20, 39). Although these receptors normally regulate their own synthesis directly by binding to ARE sequences located in their promoters, transcriptional analyses did not allow us to confirm such a point. For that reason, we decided to purify both regulators and study their capacities of binding to the ARE sequences identified.

### GST-SfbR and GST-SfbR2 bind the five ARE-containing promoters of the γ-butyrolactone gene cluster.

To confirm that the promoters containing ARE sequences were the actual targets of SfbR and SfbR2, we performed electrophoretic mobility gel shift assays (EMSAs) with glutathione *S*-transferase (GST)-SfbR or GST-SfbR2 (Fig. S6) and with DNA probes containing the five promoters with ARE sequences. The promoter of *sfb4*, which lacks an ARE-like sequence, was used as a negative control.

**FIG 7:**
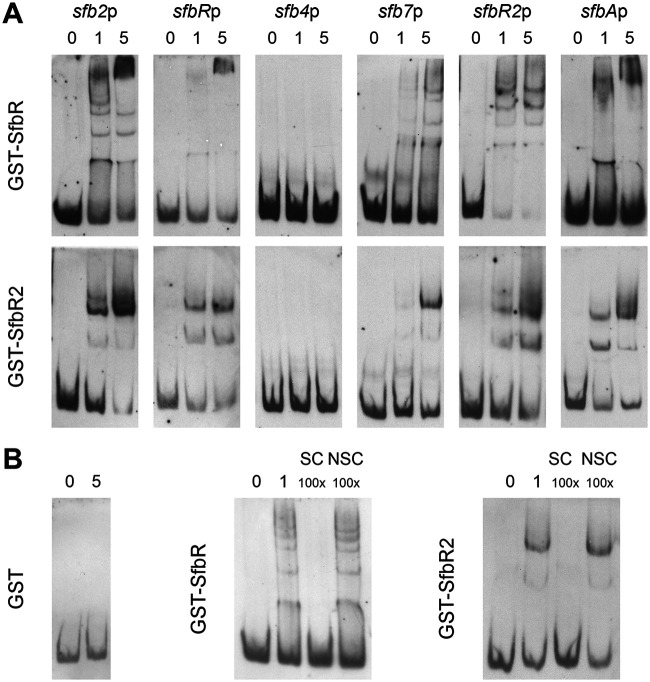
EMSAs of GST-SfbR and GST-SfbR2 binding to different promoters. (A) Promoter names are indicated above the photos. All experiments were carried out with 0.05 ng of labeled DNA probe and increasing concentrations of fusion protein (0 to 5 μM). (B) Examples of results of control reactions performed with pure GST (5 μM) and *sfb2*p and of competition experiments performed with 1 μM protein. SC, specific competitor; NSC, nonspecific competitor.

The results from the EMSAs are shown in Fig. 7. To eliminate the possibility that the interactions might have been mediated by the GST moiety of the fusion proteins, control reactions were performed under the same conditions but using pure GST instead of the fusion protein. The binding results were negative in all cases, excluding such a possibility. In the cases where bands representing retardation were observed, the intensity of the band(s) was diminished as a result of the addition of the same unlabeled DNA, suggesting that the binding was specific.

As expected, both fusion proteins retarded the DNA fragments containing the *sfb2*, *sfbR*, *sfb7*, *sfbR2*, and *sfbA* promoters, while that containing the *sfb4* promoter was not retarded, indicating that neither interacted with this region. The presence of multiple retarded bands may indicate various protein/DNA stoichiometries, the cooperative binding of monomers, and/or the binding of dimers as proposed previously for the binding of BulR1, the GBL receptor of S. tsukubaensis, to its targets (36).

### DNase I protection studies reveal that the two regulators bind the same sites.

**FIG 8:**
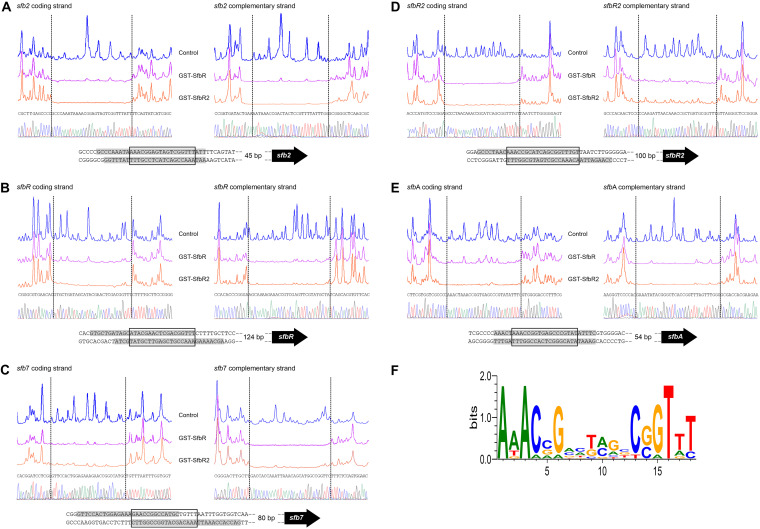
Identification of binding sites. DNase I footprints of the GST-SfbR and GST-SfbR2 proteins bound to the different promoter regions were analyzed. Promoter names are indicated above the pictures. (A to E) In each panel, the upper electropherogram (blue line) represents the control reaction. The protected nucleotide sequence is shaded in gray. Coordinates are from the translation start point. (F) Sequence logo of the nucleotide sequences that constitute SfbR and SfbR2 binding sites. The logo was constructed with the 10 protected regions observed in the footprinting assays. The height of each letter is proportional to the frequency of the base, and the height of the letter stack is proportional to the conservation quantified in bits at that position (59). The value representing total information (*R*_sequence_) for the binding site is 13.59 bits (0.76 bits per base).

To determine the precise binding sites of both regulators, we carried out DNase I footprinting assays. GST-SfbR or GST-SfbR2 protein (2 μM) was tested using 5′-end fluorescein-labeled DNA fragments. All analyses were carried out in triplicate. These analyses revealed that the two fusion proteins protected a single site in the promoters containing ARE sequences and that they bound the same sites (Fig. 8).

Assays performed with the *sfb2p* promoter region revealed a 30-nucleotide protection region in the coding strand (positions −55 to −84 with respect to the *sfb2* translation start site). In the bottom strand, the protected sequence was 28 bp long, spanning position −55 to position −82, and both regions were displaced by 2 nucleotides (Fig. 8A).

Footprinting assays of the *sfbRp* region revealed a 29-nucleotide protection region in the coding strand (positions −136 to −164 with respect to the *sfbR* translation start site). In the complementary strand the protected sequence was 30 bp long, spanning position −128 to position −157. In this case, both protected regions were displaced by 7 to 8 nucleotides (Fig. 8B).

In the case of the *sfb7* promoter, a protected region of 28 nucleotides was observed in the coding strand of *sfb7* (positions −100 to −127 from the *sfb7* translation start codon). In the bottom strand, the protected sequence was 30 bp long, at positions −83 to −112 (Fig. 8C). These protected regions were slightly displaced, i.e., 13 of their nucleotides overlapped.

Results of the analysis of the *sfbR2p* promoter region showed a protected stretch extending for 28 bp of the coding strand. This protected region was located at nucleotide positions −117 to −144 with respect to the *sfbR2* translational ATG start site. The protection region of the reverse strand was 29 nucleotides long (positions −108 to −136), with both regions being displaced by 8 to 9 nucleotides (Fig. 8D).

In the case of the *sfbA* promoter, a protected region of 28 nucleotides was observed in the coding strand of *sfbA* (positions −63 to −90 from *sfbA* translation start codon), and the same was also observed in the bottom strand (positions −63 and −90). Neither protected region was displaced (Fig. 8E).

Typically, the protected region in the sense strand of the regulated gene was accompanied by a protected region in the complementary strand, with both protected regions being slightly displaced (Fig. 8).

### Information content analysis of the SfbR and SfbR2 operators.

An information-based model of the binding site was constructed, taking into account the 10 protected regions observed in the footprinting assays. A sequence logo (59) that depicts the binding site is shown in Fig. 8F. This site spans 18 nucleotides and adjusts to the consensus AAACVGNNBVNNCSGTTT (where V represents A, C, or G; S is C or G; and B is C, G, or T). It is noteworthy that the binding site sequence displays dyad symmetry and is highly similar to consensus sequences recognized by other GBL receptors and pseudo-receptors from *Streptomyces* spp. (23, 24).

### SfbR2 is the receptor of antimycin A.

**FIG 9:**
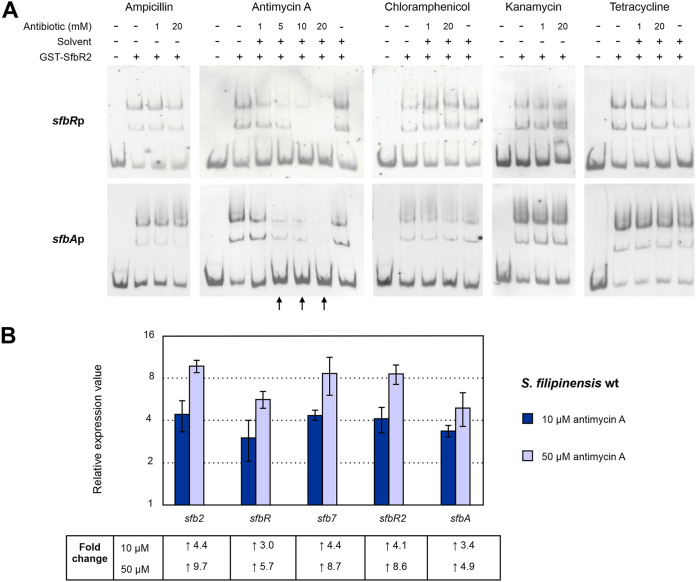
Antimycin A is an SfbR2 ligand. (A) EMSAs of GST-SfbR2 with various antibiotics. Antibiotic concentrations are indicated above each picture. Ampicillin and kanamycin were dissolved in water, while antimycin A and chloramphenicol were resuspended in ethanol and tetracycline in 0.15 M NaOH. Ethanol or 0.15 M NaOH was used as a solvent control when needed. (B) Effect of antimycin A on transcription of SfbR2 target genes *in vivo*. Antimycin A was dissolved in ethanol and added to 24-h cultures at 10 or 50 μM. Total RNA was isolated 1 h later, and expression was assessed by RT-qPCR. Transcription of each gene is expressed relative to the control (ethanol added), which was assigned a value of 1. The expression level of of *rrnA1* (encoding 16S rRNA) was used as a control. Error bars were calculated by measuring the standard deviations of the ratio values among three biological and three technical replicates of each sample. Fold change values are indicated below the panel. Primers are listed in Table 1.

SfbR2 homologues ScbR2, JadR2, and AvaR2 were previously reported to bind antibiotics as ligands (21–23). These antibiotics may be either endogenous (21) or exogenous (22). The responses of SfbR2 to different antibiotics that rendered positive results with SfbR2 homologues (chloramphenicol [JadR2] and kanamycin [AvaR2]) were analyzed by EMSAs using two DNA targets of SfbR2, the promoter regions of *sfbR* and *sfbA* (Fig. 9). Other exogenous antibiotics tested included tetracycline, other aminoglycosides such as spectinomycin or apramycin, and beta-lactams such as ampicillin (Am). The endogenous macrolide filipin and the polyketide nonribosomal peptide antimycin A commonly produced by *Streptomyces* spp. were also assayed. Dissociation of SfbR2-DNA complexes was not induced by chloramphenicol, kanamycin, spectinomycin, apramycin, tetracycline, or ampicillin, even at 20 mM concentration (Fig. 9), or filipin, which could be tested only up to a 2 mM concentration given its low solubility (not shown). In contrast, complexes were disrupted by antimycin A at a 5 mM concentration (Fig. 9). These results strongly suggest that antimycin A is recognized as a ligand by SfbR2, which in response relieves its repression of target promoters. No dissociation of SfbR-DNA complexes was observed in the presence of antimycin A, thus indicating that the GBL receptor did not bind the antibiotic and suggesting that binding of the pseudo-receptor is specific. Our findings support the concept that antibiotics may have a signaling function in *Streptomyces* and GBL pseudo-receptors serve as receptors of these signals.

To further support the idea of a role of antimycin A as an SfbR2 ligand, we performed *in vivo* assays by testing its effect on the transcription of SfbR2 target genes. S. filipinensis was grown for 24 h and added to increasing concentrations of antimycin A (or ethanol as a solvent control), and total RNA was isolated 1 h later. Transcript quantity was assessed by RT-qPCR using RNAs obtained as the template. The transcriptional levels of the genes in the presence of antimycin A were compared with those measured in its absence, which was assigned a relative expression value of 1. Interestingly, the addition of 10 μM antimycin A clearly increased transcription of every gene (Fig. 9). Moreover, transcription was further increased when we increased the antimycin A concentration to 50 μM. Taken together, these results clearly indicate that SfbR2 not only binds antimycin A but also responds to it in a concentration-dependent manner.

So far, and given that S. filipinensis genome has not been sequenced, we do not know whether it carries an antimycin A biosynthetic gene cluster. We tried to identify antimycin A in 25-fold-concentrated cell culture broth extracts using the same high-performance liquid chromatography (HPLC) method used for the detection of filipins and antimycin A as the standard (Sigma) but were not successful (see Materials and Methods), leading to the conclusion that this antifungal compound was not produced by S. filipinensis under the assay conditions used. Hence, this antibiotic could be considered to represent an exogenous antibiotic used as a signal to modulate SfbR2 DNA-binding activity. Pseudo-receptors have been described to respond to both endogenous antibiotics (21) and exogenous antibiotics (22, 23) as a way to coordinate antibiotic biosynthesis in the producing organism, and the latter seems to be the case for S. filipinensis. Future experimental studies will establish the molecular mechanism involved in this process.

### GBL regulatory model in S. filipinensis.

**FIG 10:**
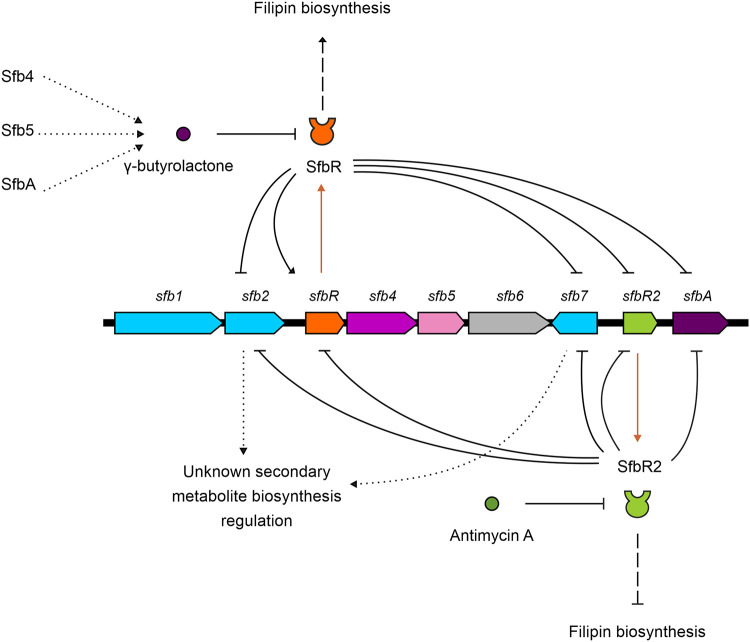
GBL regulatory model in S. filipinensis. The solid-line arrow/bar represents direct activation/repression. The dashed-line arrow/bar represents indirect activation/repression. Dotted lines represent hypothetical data. Lines in brown represent transcription and translation of *sfbR* or *sfbR2*. Gene coloring is used as described in the Fig. 1 legend.

*Streptomyces* GBLs are important signaling molecules with respect to triggering antibiotic production in a quorum sensing-dependent manner. In this work, we characterized the GBL system from S. filipinensis, finding that each of the two key players of this system, the GBL receptor and the pseudo-receptor, counteracts the transcription of the other for the modulation of filipin production. Such control over antifungal production involves an indirect effect on the transcription of filipin biosynthetic genes, presumably operating through an as-yet-unidentified regulator (Fig. 10). In this scenario, the SfbR GBL receptor acts as an activator of filipin biosynthesis whereas the SfbR2 pseudo-receptor behaves as a repressor. Whether there is a connection between this regulation of filipin biosynthesis and the recently described Pho regulation for this strain (29) remains unknown, and further studies will be required to check such a possibility.

The structure of the GBL produced by S. filipinensis is currently unknown, but by analogy of the genes found in the GBL gene cluster with those of other systems that have been characterized, it is likely synthesized by the concerted activities of GBL synthase SfbA, cytochrome P450 monooxygenase Sfb4, and nucleoside diphosphate-sugar epimerase Sfb5 (3, 16, 17, 38).

As occurs with other GBL systems, the GBL receptor and the pseudo-receptor target the same DNA sites, which are highly similar to binding sites previously identified in other GBL receptors and pseudo-receptors. Both regulators are self-regulated and repress the transcription of three other genes of the GBL cluster, specifically, the regulator-encoding genes *sfb2* (StrR family) and *sfb7* (SARP family) and the GBL synthase-encoding gene *sfbA*. SfbR2 represses its own transcription, as expected for a pseudo-receptor, but SfbR, in contrast to all the GBL receptors analyzed to date, activates its own synthesis rather than repressing it (Fig. 10).

Moreover, the SfbR2 pseudo-receptor is able to bind and respond to a presumed exogenous antibiotic, antimycin A, thus extending the number of examples indicating that antibiotics are used in *Streptomyces* species as signals to coordinate antibiotic biosynthesis in the producing organism (21–23).

The intricate regulatory network depicted here should provide important clues for understanding the regulatory mechanism governing secondary metabolism.

## MATERIALS AND METHODS

### Microbial strains and genetic procedures.

S. filipinensis DSM 40112 growth and sporulation were achieved as described elsewhere (29). Escherichia coli strain DH5α was used as a host for DNA manipulation. E. coli BL21(DE3) was used for expression studies. E. coli BW25113(pIJ790) was used for gene replacement experiments. E. coli ET12567(pUZ8002) was used as the donor in intergeneric conjugations with S. filipinensis as described previously (60). pUC19 (New England Biolabs) was used as the routine cloning vector, pSETneo (ampicillin resistance [Am^r^], neomycin resistance [Neo^r^], pUC18 replicon, ΦC31 *attP* [48]) was used for intergeneric conjugations, and pGEX-2T (GE Healthcare) was the vector used to construct expression plasmids. Plasmid DNA preparation, DNA digestion, fragment isolation, and transformation of E. coli were performed by standard procedures. The DNA probe used for genomic library (26) screening was obtained by PCR amplification of S. filipinensis chromosomal DNA with primers GBR1 and GBR2, designed against the N-terminal end of GBL receptors (Table 1), and sequenced to verify that it corresponded to the conserved helix-turn-helix domain of genuine GBL receptors. PCRs were carried out using Hybrid DNA polymerase as described by the enzyme supplier (EURx). DNA sequencing was accomplished by the dideoxynucleotide chain-termination method using a DYEnamic ET terminator cycle sequencing kit (GE Healthcare) with an Applied Biosystems ABI 3130XL DNA genetic analyzer (Foster City, CA, USA).

**TABLE 1:** Primers used in this study

Use and name	Sequence (5′→3′)^*a*^	Target (bp)
Probe for genomic library screening		
GBR1	TGGCKMRRCAGGANCGVGC	
GBR2	GAAGTGGAARTASARSGCBCCC	

Construction and verification of mutants		
SfbR-Red-F	*gtgtgggggacccgggtcagggtgaggaggtgtggcgtg*ATTCCGGGGATCCGTCGACC	
SfbR-Red-R	gtgtgtgatcgccgtgcgggtctgatccggccgtactcaTGTAGGCTGGAGCTGCTTC	
SfbR2-Red-F	*ggccacggaacggctcgggagctacggagcacctcgatg*ATTCCGGGGATCCGTCGACC	
SfbR2-Red-R	ggcagacctgcgcggtccgttgcgaaggggctgctctcaTGTAGGCTGGAGCTGCTTC	
sfbR5F	CCGCACCCGTTTCGACGCCG	
sfbR11F	GTACCGCGTCCTGATGGCCG	
sfbR12R	GGTGGTGTGTGATCGCCGTGC	
sfbR19R	GGTGTGTTCGGTCCCGTAGGCG	
sfbR2F	GCGTTCAGGGCGAGCAGGGC	
sfbR2.1F	GCGGAAGTCCAGCGTGCCC	
sfbR2R	GGCGAACGAGCAGGGTCATGG	
sfbR2.1R	TGGACAGCAGCGAGGAAGGGC	

Construction of plasmids for genetic complementation		
SfbR16F	CGACGACGAGAAATGGCGGTGG	
SfbR12R	GGTGGTGTGTGATCGCCGTGC	
SfbR2F	GCGTTCAGGGCGAGCAGGGC	
SfbR2REcoRI	GGAATTCGGCGAACGAGCAGGGTCATGG	

Construction of plasmids for protein expression		
SfbR-GST-F	TACAGGATCCGTGGCGCAGCAGGAACGGGC	
SfbR-GST-R	GGGAATTCGCCGTACTCAACCGTGCTCGAC	
SfbR2-GST-F	TACAGGATCCATGGTCAAGCAGGAACGTGC	
SfbR2-GST-R	GGGAATTCGCTGCTCTCAGCAGGTTCCC	

Analysis of cotranscription of *sfb* genes by RT-PCR		
RT-sfb12-F	GCCGCCCACCACCTGCTG	*sfb1-sfb2* (436)
sfbR2-RACE-2	GCCAGTGCCTCGACGTGCTC	
		
SfbR16F	CGACGACGAGAAATGGCGGTGG	*sfb2-sfbR* (575)
RT-sfb2R-R	GCCGCAGCGGTCAGGATCG	
		
RT-sfbR4-F	GCCCGCCTCGTCGAGCAC	*sfbR-sfb4* (391)
RT-sfbR4-R	GGTGTGTTCGGTCCCGTAGGCG	
		
RT-sfb45-F	CTGGGACGCGGTGGTGGAGGAG	*sfb4-sfb5* (657)
RT-sfb45-R	GTTGTGGTCGTGTGCGGGCTG	
		
RT-sfb56-F	GCCCGACGCCAGCAGGAAGG	*sfb5-sfb6* (358)
RT-sfb56-R	CGAGGCTGGTTTGGGTGTGGG	
		
RT-sfbR2A-F	CGGGCGTATCGGTCGAGCAG	*sfbR2-sfbA* (488)
RT-sfbR2A-R	GCGGGTGGGCTCTTGCTGGC	

Analysis of *fil* and *sfb* gene expression by RT-qPCR		
qfilA1-F	CGGCTTCCTCGACAGCATC	*filA1* (114)
qfilA1-R	GCTTCCCAGGCCAACTCC	
		
qfilA2-F	CGAGGATCTGTGGGAGTTGGTC	*filA2* (128)
qfilA2-R	CGCGGGCGTAGCTGGTC	
		
qfilR-F	AGACATGGCTCTGGAGTGTG	*filR* (82)
qfilR-R	GTGCCCACCGAACTGCTC	
		
qfilF-F	ATCCAGCAGGCGAACCAG	*filF* (125)
qfilF-R	TTGGAGAATTGACGCACCAG	
		
qfilH-F	CTCCGCCAGCTTCTACTTCC	*filH* (147)
qfilH-R	AGGGCCTCGTAGATCTTGTC	
		
qsfb2-F	GACCATGCGGATTGTCGAC	*sfb2* (78)
qsfb2-R	TAGCGGACCTCGATGGAGTC	
		
qsfbR-F	CGACGGTTTCTTTTGCTTCCC	*sfbR* (56)
qsfbR-R	ACACCTCCTCACCCTGACC	
		
qsfb7-F	CCCACAGTTCCTCCACCAG	*sfb7* (141)
qsfb7-R	AAGTACTCGGCGGCTTTGC	
		
qsfbR2-F	AGTTGTGGGCGCGTTCTG	*sfbR2* (65)
qsfbR2-R	TACGGCCACGGAGATGACAC	
		
qsfbA-F	AGGTCTTCCTCACCGGATG	*sfbA* (90)
qsfbA-R	TGCTGGTGAAGAAGGTGTGC	
		
qrrnA1-F	GACGCAACGCGAAGAACC	*rrnA1* (137)
qrrnA1-R	TGCGGGACTTAACCCAACATC	
Rapid amplification of cDNA ends (RACE)		
sfb2-RACE-1	GCTTGACGGCCAGGGACGAAC	*sfb2* TSP
sfb2-RACE-2	GCCAGTGCCTCGACGTGCTC	
sfb2-RACE-3	CGGTGATCTGGGCGTGCCTGC	
		
sfbR-RACE-1	GGGACCAGGCCAGGAAGGG	*sfbR* TSP
sfbR-RACE-2	GTCCACCTTGCCGTGCCCG	
sfbR-RACE-3	TCAGGTAGGCGAGCAGCAGGC	
		
sfb7-RACE-1	GCGGAAGTCCAGCGTGCCC	*sfb7* TSP
sfb7-RACE-2	GTCTCCAGGAGATAACCGCCCG	
sfb7-RACE-3	CGCCCCACAGTTCCTCCACC	
		
sfbR2-RACE-1	CGCACCTCCCCCACACCC	*sfbR2* TSP
sfbR2-RACE-2	ACCTCCCCCACACCCCGC	
sfbR2-RACE-3	GGGGCATCCGCTTCTCGC	
		
sfbA-RACE-1	GGACGTGCCCCTATGCCCAG	*sfbA* TSP
sfbA-RACE-2	CAGGAAGTGATGCCCCAGCGG	
sfbA-RACE-3	AAGAGACCGACCTGGCGAATGG	

EMSAs and footprinting probes		
sfb2p-EMSA-F	GCCGCCCACCACCTGCTG	*sfb2p*
sfb2-RACE-3	CGGTGATCTGGGCGTGCCTGC	
		
sfbRp-EMSA-F	CCGCACCCGTTTCGACGCCG	*sfbRp*
RT-sfb2R-R	GCCGCAGCGGTCAGGATCG	
		
sfb4p-EMSA-F	CAGCAGCGGCACGGCAAGG	*sfb4p*
sfb4p-EMSA-R	GGGGTCCACCAGCAGTCGCC	
		
sfb7p-EMSA-F	GCGTGCTGCCCAGCCGTGC	*sfb7p*
sfb7p-EMSA-R	GCACACCACCCCGGCACACG	
		
sfbR2p-EMSA-F	AGCTCGTTCCCCCTGCCCG	*sfbR2p*
sfbR2p-EMSA-R	TGGACAGCAGCGAGGAAGGGC	
		
sfbAp-EMSA-F	GAGAGCAGCCCCTTCGCAACG	*sfbAp*
RT-sfbR2A-R	GCGGGTGGGCTCTTGCTGGC	

a The sequence identical to the DNA segment upstream from the start codon of *sfbR* or *sfbR2* is in italic lowercase letters in the SfbR-Red-F sequence or the SfbR2-Red-F sequence, respectively; the sequence identical to the segment downstream from the stop codon of *sfbR* or *sfbR2* is underlined and in roman lowercase letters in the SfbR-Red-R sequence or SfbR2-Red-R sequence, respectively; the sequences identical to the *aac(3)IV* or *aadA* cassette sequence are in uppercase letters in the SfbR-Red-F and the SfbR2-Red-F sequences and in the SfbR-Red-R and SfbR2-Red-R sequences.

### Isolation of total RNA and reverse transcription-PCR.

RNA was extracted as described previously (61), and transcription was studied as indicated previously (62). Briefly, we used a SuperScript one-step reverse transcriptase PCR (RT-PCR) system with Platinum *Taq* DNA polymerase (Invitrogen) and 150 ng of total RNA as the template. Conditions were as follows: for first-strand complementary DNA (cDNA) synthesis, 50°C for 40 min followed by heating at 94°C for 2 min, amplification for 28 cycles of 94°C for 40 s and 63 to 67°C (depending of the set of primers used) for 30 s, and 72°C for 30 s. Primers (17-mers to 23-mers; Table 1) were designed to detect the possible cotranscription of neighboring genes. Negative controls were carried out with each set of primers and with Platinum *Taq* DNA polymerase in order to confirm the absence of contaminating DNA in the RNA preparations. The identity of each amplified product was corroborated by direct sequencing of the PCR product.

### Reverse transcription-quantitative PCR.

Reverse transcription of total RNA was performed on selected samples with 5 μg of RNA and 12.5 ng/μl of random hexamer primer (Invitrogen) using SuperScript III reverse transcriptase (Invitrogen) as described previously (62). Reactions were carried out on three biological replicates with three technical replicates each, and appropriate controls were included to verify the absence of genomic DNA (gDNA) contamination in RNA and primer-dimer formation. Primers (Table 1) were designed to generate PCR products corresponding to the region between 56 and 147 bp, near the 5′ end of mRNA. The PCRs were initiated by incubating the sample at 95°C for 10 min followed by 40 cycles at 95°C for 15 s and 64 to 70°C (depending of the set of primers used) for 34 s. To check the specificity of real-time PCRs, a DNA melting curve analysis was performed by holding the sample at 60°C for 60 s followed by slowly ramping up the temperature to 95°C. Baseline and threshold values were determined by the use of StepOnePlus software. Threshold cycle (*C_T_*) values were normalized with respect to *rrnA1* mRNA (encoding 16S rRNA). Relative changes in gene expression were quantified using the Pfaffl method (63) and REST software (64). The corresponding real-time PCR efficiency (E) of one cycle in the exponential phase was calculated according to the equation E = 10^[−1/slope]^ (65) using 5-fold dilutions of genomic DNA ranging from 0.013 to 40 ng (*n* = 5 or 6 with three replicates for each dilution) with a coefficient of determination *R*^2^ value of >0.99.

### Rapid amplification of cDNA ends.

Transcription start points were identified by using a 5′ RACE kit (Invitrogen) in accordance with the manufacturer’s instructions (version 2.0) and as described previously (61), using 5 μg of total RNA for first-strand cDNA synthesis and the gene-specific primers listed in Table 1.

### Assessment of filipin and antimycin A production.

Filipin and antimycin A production was assessed after growth at 30°C in YEME medium without sucrose. To assay filipin in culture broths, 1 volume of culture was extracted with 1 volume of methanol and was further diluted with methanol to bring the absorbance at 338 nm into the range of 0.1 to 0.4 units. Solutions of pure filipin III (Sigma) were used as controls. The identity of filipin was confirmed by analysis of its UV-visible absorption spectrum (absorption peaks at 356, 338, 320, and 311 nm). Quantitative determination of filipin was performed as previously described (26), using a Mediterranea Sea C_18_ column (Teknokroma) (4.6 by 150 mm; particle size, 3 mm). For antimycin A production assessment, 1 volume of culture was extracted with 2 volumes of ethyl acetate and was dried by rotary evaporation. The pellet was then resuspended in methanol prior to HPLC analysis. The same chromatographic method was used for estimation of antimycin A production at 318 nm. Pure antimycin A (Sigma) was used as the standard.

### Construction of mutants.

Deletion of *sfbR* from the S. filipinensis chromosome was performed by replacing the wild-type gene with a cassette containing an apramycin-selective marker using a PCR-based system (66). Plasmid pIJ773, containing an apramycin resistance gene [*aac(3)IV*] and an *oriT* replication origin, was used as a template. The mutant was constructed using oligonucleotides SfbR-Red-F and SfbR-Red-R (Table 1) as the forward and reverse primers, respectively (in that table, the sequence identical to the DNA segment upstream from the start codon of *sfbR* is in lowercase italics and the sequence identical to the segment downstream from the stop codon of *sfbR* is underlined and in lowercase roman). These two long PCR primers were designed to produce a deletion of *sfbR* just after its start codon, leaving only its stop codon behind. The 3′ sequence of each primer matches the right or left end of the disruption cassette (the sequence is shown in uppercase characters in both primers). The extended resistance cassette was amplified by PCR, and E. coli BW25113(pIJ790) bearing cosmid 8H10 was electrotransformed with this cassette. The isolated mutant cosmid was introduced into nonmethylating E. coli ET12567 containing RP4 derivative pUZ8002. The mutant cosmid was then transferred to S. filipinensis by intergeneric conjugation. Double-crossover exconjugants were screened for apramycin resistance followed by confirmation by PCR. A similar strategy was used for the deletion of *sfbR2* but using plasmid pIJ778 containing the spectinomycin/streptomycin resistance gene (*aadA*) and *oriT* as the template and primers SfbR2-Red-F and SfbR2-Red-R (Table 1). In this case, double-crossover exconjugants were screened for spectinomycin resistance.

### Construction of plasmids for gene complementation.

In order to complement the *sfbR* replacement mutant, a 1,261-bp DNA fragment containing the entire *sfbR* gene plus its promoter region was amplified by PCR with primers SfbR16F and SfbR12R (Table 1) using S. filipinensis chromosomal DNA as the template. The PCR product was cloned into EcoRV-cut pSETneo (47) to yield pSETneo::sfbR.

Similarly, for S. filipinensis Δ*sfbR2* gene complementation, a 1,489-bp DNA fragment containing the *sfbR2* gene plus its promoter was amplified by PCR with primers SfbR2F and SfbR2REcoRI (Table 1). The PCR product was cloned into EcoRI/EcoRV-cut pSETneo to yield pSETneo::sfbR2.

### Construction of plasmids for protein expression.

The SfbR gene was amplified for insertion into GST expression vector pGEX-2T using PCR. The forward primer used, SbRF-GST-F, introduced a unique BamHI site at the 5′ end of the gene, while the reverse primer, SbR-GST-R, carried an EcoRI site 7 nucleotides downstream from the TGA translational stop codon (Table 1). The amplified DNA fragment was digested with BamHI and EcoRI and cloned into the same sites of pGEX-2T to generate pGEX-2T::sfbR. The amplified DNA fragment was sequenced from the expression vector in order to eliminate any errors introduced by the DNA polymerase. Similarly, SfbR2 was amplified using forward primer SbR2-GST-F and reverse primer Sb2R-GST-R (Table 1). Cloning of the amplified and digested DNA fragment into pGEX-2T yielded pGEX-2T::sfbR2.

### Expression and purification of GST fusion proteins.

E. coli BL21(DE3) cells containing pGEX-2T::sfbR or pGEX-2T::sfbR2 were grown at 30°C in 100 ml LB medium containing 100 μg/ml of ampicillin to an optical density at 600 nm (OD_600_) of 0.5 and induced by adding isopropyl 1-thio-β-d-galactopyranoside (IPTG) to reach a final concentration of 0.1 mM, and then growth was permitted to continue for an additional 5 h at 22°C. Cells were harvested, resuspended in phosphate-buffered saline (PBS; 140 mM NaCl, 2.7 mM KCl, 10 mM Na_2_HPO_4_, 1.8 mM KH_2_PO_4_, pH 7.3), and lysed by sonication using an ultrasonic processor Sonifier B-12 apparatus (Branson Inc.). The insoluble material was separated by centrifugation, and the soluble fraction was applied to a GSTrap HP (GE Healthcare) column. Proteins were eluted with 10 mM reduced glutathione–50 mM Tris-HCl (pH 8.0) and conserved in 20% glycerol at −80°C before use. Protein elution was quantified using Bradford reagent, and the presence of the fusion protein was assessed by SDS-PAGE (see Fig. S6 in the supplemental material).

### DNA-protein binding assays.

DNA-binding tests were performed by electrophoretic mobility shift assay (EMSA). The DNA fragments used for EMSA were amplified by PCR using the primers listed in Table 1 and S. filipinensis genomic DNA as the template, sequenced to confirm the absence of any mutations, and then labeled at both ends with digoxigenin (DIG) using a DIG oligonucleotide 3′-end labeling kit (Roche Applied Science) (2nd generation).

A standard binding reaction mixture contained 0.005 ng/μl labeled DNA probe, 100 mM HEPES (pH 7.6), 10 mM KCl, 5 mM MgCl_2_, 1 mM dithiothreitol (DTT), 1% Tween 20, 7.8 mM glutathione, 40 μg/ml poly[d(I·C)], and 5% glycerol in a 10-μl final volume. Reaction mixtures were incubated at 30°C for 10 min and then loaded onto 5% polyacrylamide (29:1) native gels in 0.5× Tris-borate-EDTA (TBE) buffer. After electrophoresis, DNA was electroblotted onto a nylon membrane (HyBond-N; Amersham Biosciences) in 0.5× TBE buffer. The DNA was fixed by UV cross-linking, detected with antidigoxigenin antibodies, and developed by chemiluminescence performed with CDP-Star reagent (Roche Applied Science).

When EMSAs were performed with the addition of antibiotics, antibiotic was added after 10 min preincubation of the probe with the protein (1 μM) and then further incubated for 10 min before loading onto polyacrylamide gels.

### Footprinting assays.

DNase I footprinting assays were performed by the fluorescent labeling procedure as described previously (67), using GST-SfbR or GST-SfbR2 proteins. The DNA fragments used were the same as those used for the EMSAs and were cloned into pUC19 and amplified by PCR using the universal and reverse primers, with one of them labeled with 6-carboxyfluorescein. The PCR products were purified after agarose gel electrophoresis, and DNA concentrations were determined with a NanoDrop ND-1000 spectrophotometer (Thermo Scientific).

For footprinting, 0.42 pmol of labeled DNA fragment was incubated with GST-SfbR or GST-SfbR2 protein using the same conditions used for the gel shift assays. Lyophilized bovine pancreas DNase I (Roche grade I) was reconstituted in a reaction mixture containing 20 mM Tris HCl (pH 7.0), 50 mM NaCl, 100 μg/ml bovine serum albumin (BSA), 1 mM DTT, and 10% glycerol to achieve a final concentration of 2.5 × 10^−3^ units/μl. Nuclease digestions were carried out with 7.5 × 10^−3^ units at 30°C for 1 min and stopped with 180 μl of 40 mM EDTA in 9 mM Tris-HCl (pH 8.0). After phenol-chloroform purification and ethanol precipitation, samples were loaded in an Applied Biosystems ABI 3130 DNA genetic analyzer (Foster City, CA, USA). Results were analyzed with the GeneMarker program (SoftGenetics).

### Bioinformatic analysis.

We used ORFfinder and BLASTp at the NCBI server for ORF identification and protein annotation, respectively, and the translate tool at ExPASy for translation. The matrices used to search for the regions at positions −35 and −10 were those derived from the alignments of class C and class A promoters previously described by Bourn and Babb (57). To search for a combination of “class C–*n* nucleotides of separation–class A,” we included *n* columns of null values in the combined matrix. To obtain the logos of the binding sites of the SfbR and SfbR2 regulators, we used the BiPad server (68). Phylogenetic analyses were performed using software package MEGA version 6 (69). Distance analysis was performed using the neighbor-joining method according to the two-parameter model. The robustness was quantified by the use of a bootstrap test with 1,000 replicates.

### Accession number(s).

The sequence of the GBL cluster has been deposited in the GenBank database under accession number MT017918.

## Supplementary Material

Supplemental file 1
