# Supplementary material for: The Streptomyces filipinensis Gamma-Butyrolactone System Reveals Novel Clues for Understanding the Control of Secondary Metabolism
Source: Appl Environ Microbiol. 2020 Sep 1;86(18):e00443-20. doi: 10.1128/AEM.00443-20 (PMC7480387; doi:10.1128/AEM.00443-20)
Supplement: Supplemental file 1 [file AEM.00443-20-s0001.pdf]

Supplementary information for:

The gamma-butyrolactone system from *Streptomyces filipinensis* reveals novel clues to understand secondary metabolism control.

Eva G. Barreales,[a] Tamara D. Payero,[a] Ester Jambrina,[a] Jesús F. Aparicio[a#]

Table S1. Deduced function of each gene of the GBL cluster in *S. filipinensis* and orthologous counterparts in other bacterial species.

ORF	Size (pb)	Size (aa)	Predicted function	ID (%)	Protein/Organism
sfb1	1950	649	SARP-like regulator	39<br>35	AfsR *S. coelicolor* (BAA14186)<br>DnrI *S. peucetius* (AAA26736)
sfb2	1050	349	StrR-like regulator	49<br>42	KasT *S. kasugaensis* (BAC53615)<br>StrR *S. griseus* (CAH94333)
sfbR	702	233	TetR-like regulator	57<br>56	SpbR *S. pristinaespiralis* (AYO26762)<br>AvaR1 *S. avermitilis* (AKK07686)
sfb4	1278	425	P450 monooxygenase	57<br>56	Orf16* *S. fradiae* (AAD40802)<br>Cyp *S. avermitilis* (BAC71416)
sfb5	846	281	Dehydrogenase	51<br>50	Orf4 *Streptomyces* sp. SBI034 (AKP54263)<br>ScbC *S. coelicolor* (CAB60186)
sfb6	1482	493	Hypothetical protein	54<br>35	Hypothetical protein *S. virginiae* (WP_051762480)<br>SCO0249 *S. coelicolor* (CAB53289)
sfb7	828	275	SARP-like regulator	66<br>65	BulY *S. tsukubaensis* (CBY91988)<br>FarR4 *S. lavendulae* (BAG71714)
sfbR2	618	205	TetR-like regulator	51<br>47	PapR5 *S. pristinaespiralis* (CBW45767)<br>SgvR1 *S. griseoviridis* (AGN74903)
sfbA	1014	337	AfsA-like protein	57<br>55	SrrX *S. rochei* (BAC76543)<br>Lct9 *S. rishiriensis* (ABX71092)

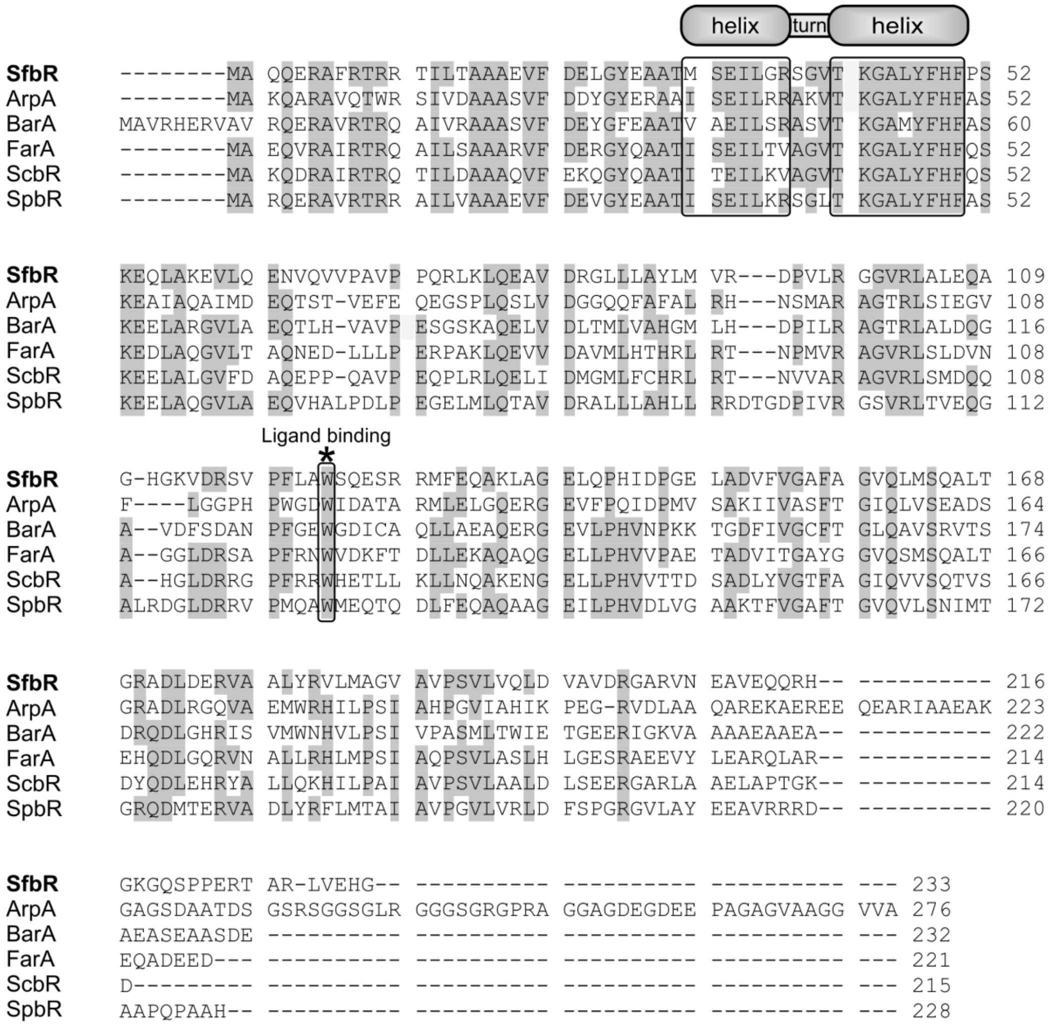

Fig S1. Alignment of *S. filipinensis* SfbR with other GBL receptors. Orthologues are ArpA from *S. griseus* (BAA36282), BarA from *S. virginiae* (BAA36282), FarA from *S. lavendulae* (BAG71716), ScbR from *S. coelicolor* (CAB60184) and SpbR from *S. pristinaespiralis* (AAK07686). Identical amino acids in at least four of the six sequences are shaded. The amino acid residues that form the helix-turn-helix motif for DNA binding are indicated and the conserved tryptophan involved in GBL binding (W123 in *S. filipinensis*) is boxed.

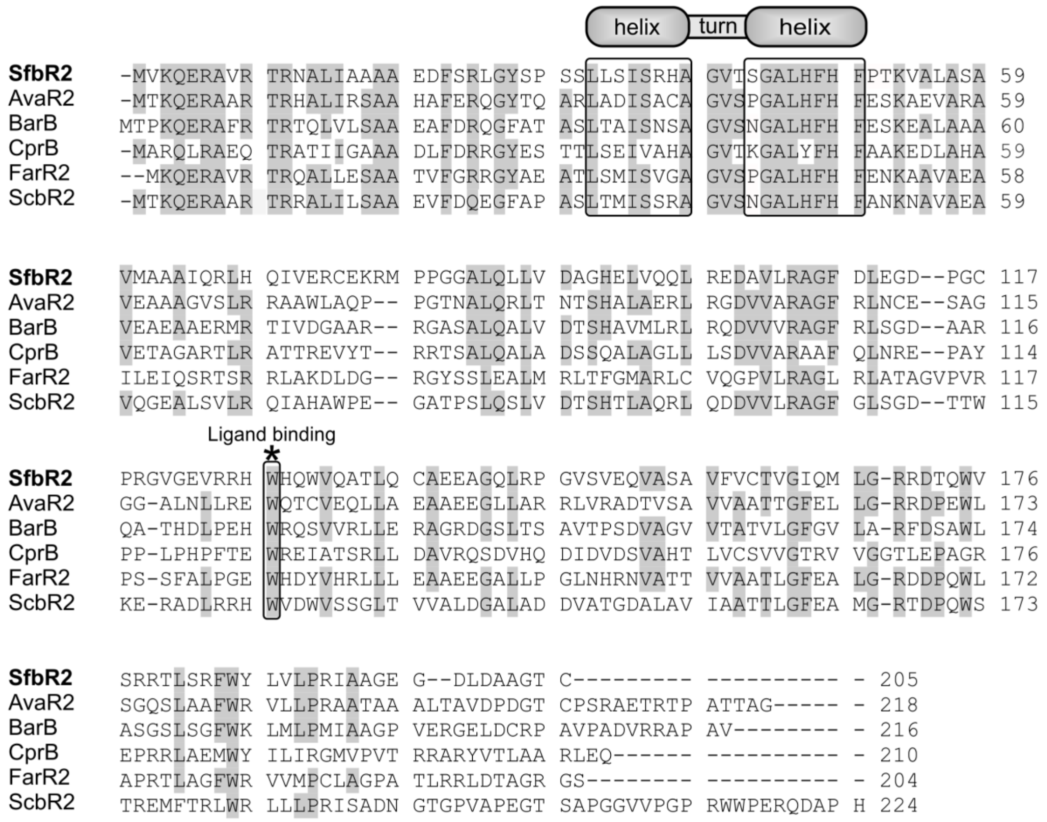

Fig S2. Alignment of *S. filipinensis* SfbR2 with other GBL pseudo-receptors. Orthologues are AvaR2 from *S. avermitilis* (BAC71414), BarB from *S. virginiae* (BAA23612), CprB from *S. coelicolor* (BAA28748), FarR2 from *S. lavendulae* (BAG74711) and ScbR2 from *S. coelicolor* (CAC37887). Identical amino acids in at least four of the six sequences are shaded. The amino acid residues that form the helix-turn-helix motif for DNA binding are indicated and the conserved tryptophan likely to be involved in ligand binding (W128 in *S. filipinensis*) is boxed.

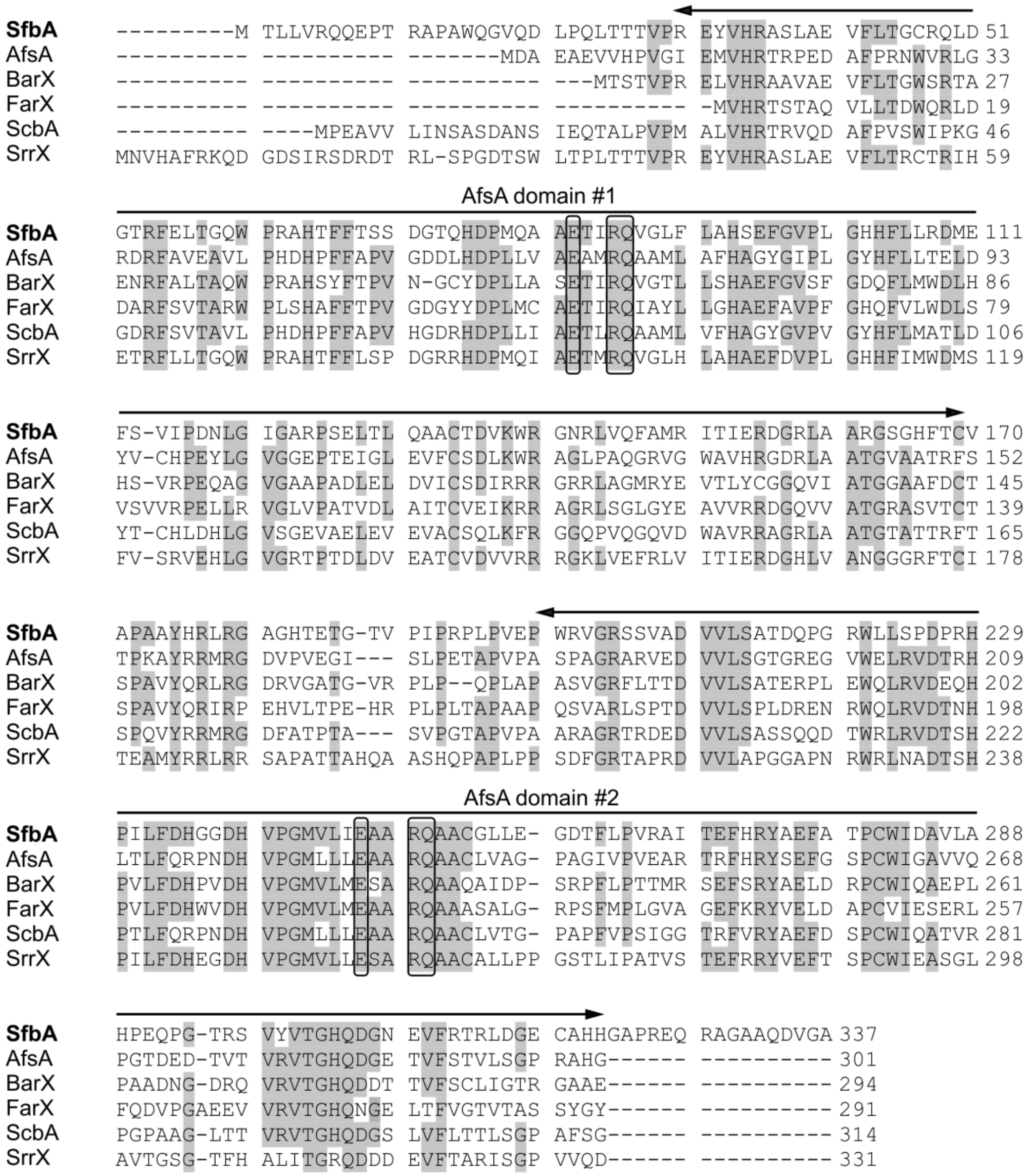

Fig S3. Alignment of *S. filipinensis* SfbA with other GBL synthases. Orthologues are AfsA from *S. griseus* (BAH47547), BarX from *S. virginiae* (BAA23611), FarX from *S. lavendulae* (BAA21858), ScbA from *S. coelicolor* (CAA07627) and SrrX from *S. rochei* (BAC76543). Identical amino acids in at least four of the six sequences are shaded. The amino acid residues likely to be involved in catalysis (E83, R86, Q87 and E247, R250, Q251 in *S. filipinensis*) are boxed. The two AfsA domains characteristic of these proteins (Pfam03756) are indicated.

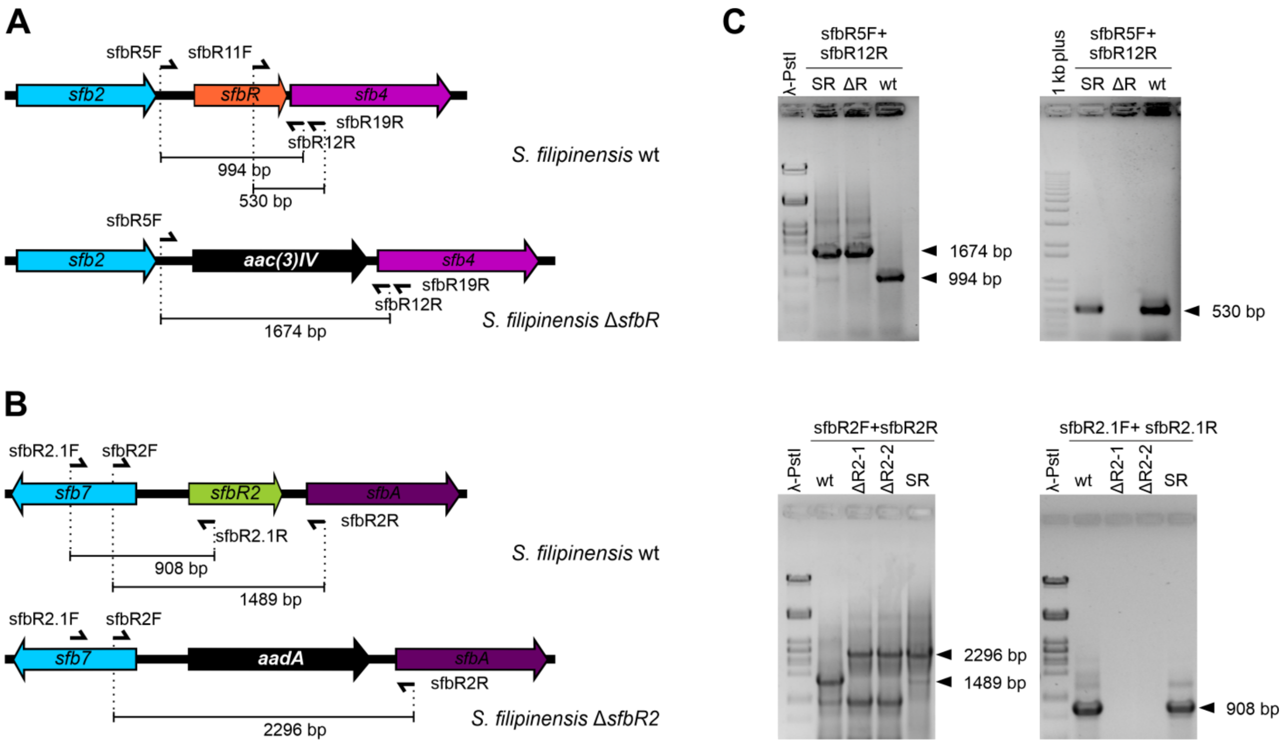

Fig S4. Construction of *sfbR* and *sfbR2* mutants. A) Predicted PCR fragment amplification of the parental strain and the *ΔsfbR* mutant. The primers used in the assay are indicated with arrowheads. The *acc(3)IV-oriT* cassette is indicated in black. B) Predicted PCR fragment amplification of the parental strain and the *ΔsfbR2* mutant. The primers used in the assay are indicated with arrowheads. The *aadA* cassette is indicated in black. C) PCR analyses of the wild type and the mutants. PCR fragments were verified by Sanger sequencing. SR: simple recombinant, ΔR: Δ*sfbR*; ΔR2: Δ*sfbR2*.

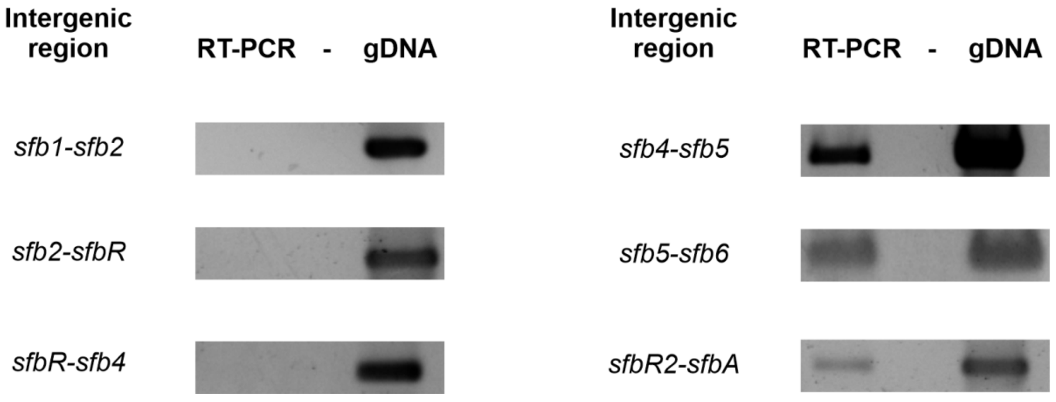

Fig S5. RT-PCR amplification of the *sfb* cluster intergenic regions. Total RNA was prepared after growth for 48 h in YEME medium without sucrose. In the PCR 40 amplification cycles were used to detect low abundant transcripts. Primers, designed to obtain a cDNA corresponding to unabated transcription between the two genes, are listed in Table 1.

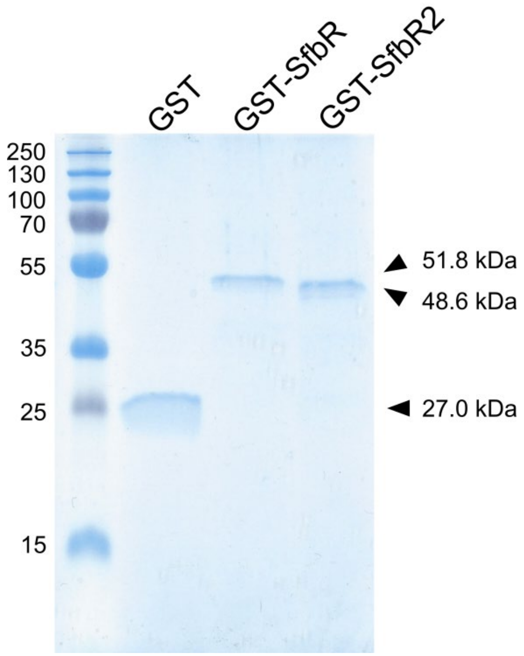

Figure S6. Purified GST fusion proteins. 2 µg purified GST, GST-SfbR and GST-SfbR2 proteins after affinity chormatography were analysed by SDS-PAGE. Left lane, molecular size markers (in kDa).
